# Establishment of ^68^Ga-DOTA-Based Pretargeted Radioimmunodiagnosis

**DOI:** 10.1021/acs.molpharmaceut.5c01766

**Published:** 2026-05-19

**Authors:** Darren R. Veach, Daniela Burnes Vargas, Baharul Islam, Sang Gyu Lee, Leah Gajecki, Brett A. Vaughn, Guangbin Yang, Teja Muralidhar Kalidindi, Naga Vara Kishore Pillarsetty, Niloufar Salehi, Ambika P. Jaswal, Sayani Saha, Alexandre B. Le Roux, Hong Xu, Hong-fen Guo, Ouathek Ouerfelli, Nai-Kong V. Cheung, Simone Krebs, Steven M. Larson, Sarah M. Cheal

**Affiliations:** † Department of Radiology, 5803 Memorial Sloan Kettering Cancer Center, New York, New York 10065, United States; ‡ Program in Molecular Pharmacology, 12295 Memorial Sloan Kettering Cancer Center, New York, New York 10065, United States; § Organic Synthesis Core Facility, 132189 Memorial Sloan Kettering Cancer Center, New York, New York 10065, United States; ∥ Department of Pediatrics, Memorial Sloan Kettering Cancer Center, New York, New York 10065, United States; ⊥ Department of Radiology, Weill Cornell Medicine, New York, New York 10021, United States; # Department of Nuclear Medicine, The University of Texas MD Anderson Cancer Center, Houston, Texas 77030, United States; ∇ Molecular Imaging Innovations Institute, Department of Radiology, Weill Cornell Medicine, New York, New York 10021, United States

**Keywords:** GPA33, radioimmunodiagnosis, gallium-68, pretargeted, bispecific antibody

## Abstract

DOTA-radiometal (DOTA: 1,4,7,10-tetraazacyclododecane-1,4,7,10-tetraacetic acid) hapten complexes of β-emitters (^177^Lu or ^90^Y) and α-emitters (^225^Ac) can be selectively targeted to tumors using an antitumor/anti-DOTA bispecific antibody (BsAb) approach in pretargeted radioimmunotherapy (DOTA-PRIT). Clinical translation of DOTA-PRIT may be accelerated by the development of a ^68^Ga companion diagnostic for PET imaging. Here, we report a novel radiohapten, which uses a 1,4,7-triazacyclononane,1-glutaric acid-4,7-acetic acid (NODAGA) chelator for ^68^Ga labeling while retaining ^175^Lu in a benzyl-DOTA moiety for high-affinity antibody recognition (NODAGA-Pr; Pr = Proteus). Radiolabeling of NODAGA-Pr with ^68^Ga showed high radiochemical yield and purity, and *in vitro* studies confirmed high radiostability and negligible serum-protein binding. *In vivo*, using a DOTA-PRIT system targeting the GPA33 antigen in a human colorectal cancer mouse model, [^68^Ga]­Ga-NODAGA-Pr demonstrated efficient tumor uptake and high-contrast PET imaging. Additional studies using an established DOTA-hapten test system—human embryonic kidney 293T cells expressing a transmembrane-anchored anti-DOTA scFv huC825 further confirmed its *in vivo* PRIT performance. These findings support [^68^Ga]­Ga-NODAGA-Pr as a promising agent for pretargeted immunoPET and for imaging engineered cells expressing a radiohapten capture reporter gene, with favorable pharmacokinetics and tumor targeting well matched to existing therapeutic DOTA-radiohaptens.

## Graphical abstract

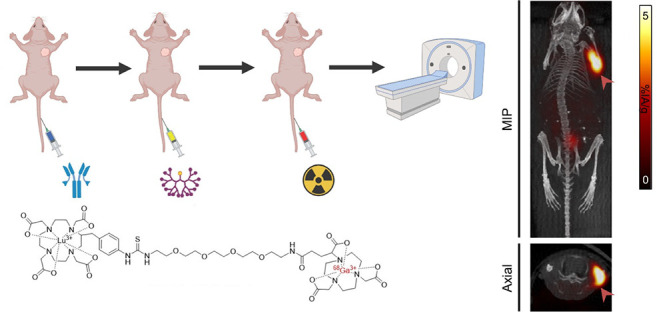

## Introduction

Pretargeted radioimmunodiagnostics and radioimmunotherapy are active fields of research in the development of novel cancer radiotheranostics.^1^ Unlike conventional approaches that rely on direct administration of radioimmunoconjugates (“single-step”), pretargeting strategies (“multi-step targeting”) involve the sequential administration of a tumor-targeting antibody followed by a radiolabeled small molecule (radiohapten or complementary radioligand), allowing for highly specific tumor targeting while minimizing off-target uptake.^1^ We recently developed a bispecific antibody (BsAb) approach for pretargeted radioimmunotherapy (PRIT), employing an ultrahigh-affinity anti-DOTA single-chain variable fragment known as “C825”^2^ to selectively deliver α- and β-emitting radioisotopes to a variety of human solid tumors—a method referred to as DOTA-PRIT.

Established small-molecule DOTA-haptens used in DOTA-PRIT include *S*-2-(4-aminobenzyl)-1,4,7,10-tetraazacyclododecane tetraacetic acid (*p*-NH_2_-Bn-DOTA), which is suitable for labeling with ^177^Lu and ^86^Y, and a specialized molecule “Proteus” (Pr), designed for use with ^111^In and ^225^Ac for image-guided α-PRIT.^3^ The Pr hapten comprises a nonradioactive ^175^Lu-benzyl-DOTA complex—recognized by the C825 antibody—a tetraethylene glycol (PEG_4_) linker, and an empty 1,4,7,10-tetraazacyclododecane-1,4,7-triacetic acid (DO3A) for chelating therapeutic or diagnostic radiometals.^3^ These radiohaptens exhibit highly favorable pharmacokinetic and biodistribution properties for pretargeting applications, characterized by favorable *in vivo* stability, rapid renal clearance, minimal hepatobiliary involvement, and exceptionally low whole-body retention.^3,4^

As we continue to optimize and expand the versatility of DOTA-PRIT, we aim to leverage the unique advantages of theranostic radiopharmaceuticals—particularly their tremendous value in understanding drug behavior and quantifying normal organ and tumor exposure using noninvasive imaging. This capacity empowers patient selection, personalized treatment planning and patient selection and dosimetry.^5^ In this study, we extend our diagnostic PET toolkit beyond esoteric ^86^Y-radiohaptens.

Clinically, ^68^Ga (half-life = 1.1 h)-based PET imaging probes are widely used for diagnosis, staging, and monitoring of treatment response.^6^ The popularity of ^68^Ga stems from its availability from a generator synthesis and its coordination chemistry as a trivalent radiometal, which allows for facile, efficient, and stable complexation with a wide range of well-characterized chelators such as DOTA and 1,4,7-triazacyclononane, 1-glutaric acid-4,7-acetic acid (NODAGA).^7^ FDA-approved probes include [^68^Ga]­Ga-PSMA-11 (targeting prostate-specific membrane antigen in prostate cancer) and [^68^Ga]­Ga-DOTATATE (targeting somatostatin receptors in neuroendocrine tumors).

While short-lived diagnostic isotopes like ^68^Ga are typically used with peptides—due to their pharmacokinetics aligning with ^68^Ga’s short physical half-life—DOTA-PRIT leverages similar small-molecule kinetics resulting in exceptionally rapid tumor targeting, making it feasible to use ^68^Ga in pretargeted immunoPET. In this approach, tumor-localized BsAbs capture the radiohapten within 1 h postinjection, well-matched to ^68^Ga’s physical decay. This makes ^68^Ga an ideal isotope for pretargeted PET imaging.

To explore the potential of extending the DOTA-PRIT platform to^68^Ga-based PET diagnostics, we investigated the use of ^68^Ga-labeled DOTA-haptens (^68^Ga-radiohaptens) as potential companion imaging agents. Specifically, we evaluated *in vivo* DOTA-PRIT performance of ^68^Ga-labeled Pr^3^ ([^68^Ga]­Ga-Pr) and ^68^Ga-labeled NODAGA-Pr ([^68^Ga]­Ga-NODAGA-Pr) using an established 3-step DOTA-PRIT regimen against glycoprotein A33 (GPA33) tumor antigen. GPA33 is expressed in over 95% of human primary and metastatic colorectal cancers^8^ and is a well-validated target for both radioimmunodiagnosis and radioimmunotherapy using radiolabeled forms of the anti-GPA33 monoclonal antibody huA33.^9–12^ To further characterize the *in vivo* properties of our novel ^68^Ga-radiohaptens, we tested them in a previously established engineered cell-based test system for radiohaptens in pretargeted imaging and radioimmunotherapy applications: human embryonic kidney 293T cells expressing transmembrane-anchored humanized C825 antibody (293T-huC825).^13^

Building on the success of [^111^In]­In-Pr^3^ and [^225^Ac]­Ac-Pr^3^, we hypothesized that [^68^Ga]­Ga-Pr and [^68^Ga]­Ga-NODAGA-Pr would serve as ideal ^68^Ga-radiohaptens for high-contrast pretargeted radioimmunodiagnosis, owing to their favorable physicochemical characteristics—particularly their small size and low lipophilicity. These studies aim to establish a novel ^68^Ga-radiohapten for ^68^Ga-DOTA-based pretargeted radioimmunodiagnosis of GPA33-expressing human colorectal cancer (CRC) and to provide a suitable diagnostic companion for future theranostic applications involving radiohapten capture.^14^

## Materials and Methods

### Synthesis of Radiohapten Precursor NODAGA-Pr

Chemicals were of reagent grade and were used as received, without further purification. The bifunctional chelators (*S*)-*p*-SCN-Bn-DOTA and NH_2_-PEG_4_-NODAGA were purchased from Macrocyclics and CheMatech, respectively. For synthesis of the novel NODAGA-Pr, *p*-SCN-Bn-DOTA·Lu^3+^ major isomer complex (20 mg, 27.6 μmol) and NH_2_-PEG_4_-NODAGA (17 mg, 28.6 μmol) were dissolved in anhydrous dimethylformamide (DMF) (0.8 mL) before treatment with triethylamine (Et_3_N) (20 μL). The resulting mixture was stirred at room temperature overnight. Solvents were then removed by vacuum evaporation, and the colorless residue was purified by reverse phase (RP) C-18 high-performance liquid chromatography (HPLC), using the gradient 5–40% acetonitrile in water (both contained 0.05% v/v trifluoroacetic acid).

### General Radiochemistry Considerations

Radiochemistry was performed in appropriately shielded chemical fume hoods equipped with electronic flow monitoring and sliding leaded glass windows. A CRC-55tR dose calibrator was used to measure radioactivity using manufacturer recommended calibration settings (Capintec Inc., Florham Park, NJ). Buffers and water used for radiochemical synthesis were treated with 5% w/v Chelex ion-exchange resin (BT Chelex 100 Resin, Bio-Rad Inc., Hercules, CA) to remove adventitious heavy metals. Plasticware (pipet tips and microcentrifuge tubes) were trace metal grade/RNA grade.

The identity and the radiochemical purity (RCP) of labeled products were determined with radioHPLC and/or radio-thin layer chromatography (radioTLC). RadioHPLC was performed on a Shimadzu Prominence HPLC system comprised of an LC-20AB dual pump module, DGU-20A3R degasser, SIL-20ACHT autosampler, SPD-20A UV–vis detector and a Bioscan Flow-Count B-FC-1000 with PMT/NaI radioactivity detector in-line. Separations were run on an analytical 4.6 × 250 mm Gemini-NX C18 or Fusion RP C18 HPLC column (Phenomenex, Inc., Torrance, CA). Unless otherwise specified, HPLC conditions were as follows: solvent A: 10 mM NH_4_OAc (pH 5.0); solvent B: CH_3_CN; flow rate: 1.0 mL/min; λ = 254 nm; injection volume: 10–50 μL; gradient: 0% B to 40% B over 10 min. Samples of free radiometals, reaction mixtures, and purified products were diluted 1:5 in 5 mM diethylenetriamine pentaacetate (DTPA) prior to analysis.

For radioTLC, aliquots (1 μL) of the crude reaction mixtures were spotted on glass microfiber chromatography paper impregnated with silica gel (iTLC-SG, Agilent Technologies), developed in 0.1 M sodium citrate buffer (pH 5.0), and scanned using an Eckert & Ziegler iTLC scanner (AR-2000). Under these conditions, [^68^Ga]­Ga-NODAGA-Pr remains at baseline, while [^68^Ga]­Ga-DTPA migrates to an *R*_*f*_ of 1.0. Additional radioTLC was performed using a mobile phase of 1.25 M ammonium acetate buffer (pH 5.5): DMF (1:1) as the mobile phase to assess the presence of [^68^Ga]­Ga-colloid. In this system, [^68^Ga]­Ga-colloid remains at the baseline, whereas [^68^Ga]­Ga-NODAGA-Pr or [^68^Ga]­Ga-Pr migrate to an *R*_*f*_ of 1.0.

### ^68^Gallium Labeling of NODAGA-Pr and Pr

[^68^Ga]­GaCl_3_ (185 MBq/5.0 mCi) in 0.5–1 mL 0.1 M HCl was eluted from a GalliaPharm ^68^Ge/^68^Ga generator (Eckert & Ziegler Radiopharma GmbH, Berlin, Germany) and transferred to a metal-free 2 mL microcentrifuge tube. A typical [^68^Ga]­Ga-Pr radiosynthesis is as follows (note: an identical procedure is used for [^68^Ga]­Ga-NODAGA-Pr). To 700 μL of 1 M sodium acetate buffer (pH 5.0) in a 1.5 mL Eppendorf Safe-Lock tube (Biopur grade), [^68^Ga]­GaCl_3_ (159 MBq/4.3 mCi in 500 μL 0.1 M HCl) was added, followed by 4.3 μL of Pr solution (4.3 nmol in water). The mixture was gently mixed and heated at 95 °C for 15 min in an Eppendorf ThermoMixer. The reaction was quenched with 4.3 μL of 10 mM DTPA and analyzed by radioTLC.

Initially, a mass titration study was performed to determine the maximum achievable molar activity while maintaining high RCP and radiochemical yield (i.e., both >90%). A NODAGA-Pr stock solution of 0.1 nmol/μL in water was used. Volumes corresponding to 10, 5, 2, 1, and 0.2 nmol of NODAGA-Pr construct were pipetted into five PCR grade polypropylene microfuge tubes. Chelexed 0.5 M NaOAc pH 5.3 buffer was added to bring the total volume to 200 μL. To this was added 200 μL of [^68^Ga]­GaCl_3_ in 0.1 M HCl (37 MBq/1.0 mCi) as eluted from the generator; therefore, the final concentrations of NODAGA-Pr were 25, 12.5, 5, 2.5, and 0.5 μM in a volume of 400 μL. The tubes were mixed and placed in an 80 °C heat block for 10 min, cooled for 2 min and then quenched with the addition of 10 μL 50 mM aqueous EDTA. Aliquots of the crude labeling reactions (200 μL) were transferred into HPLC autosampler vials and analyzed by radioHPLC.

### Radiolabeling Procedure for Animal Studies

A typical radiolabeling procedure was performed as follows: NODAGA-Pr (2 nmol in 20 μL water) was added to neutralized [^68^Ga]­GaCl_3_ (175 MBq/4.7 mCi) and mixed gently (final NODAGA-Pr concentration ∼2 μM). The tube was placed in a heat block at 95 °C for 15 min. After cooling for 5 min, the entirety was gravity loaded on a 30 mg Strata-X SPE cartridge (Phenomenex, Torrance CA), which had been equilibrated with 1 mL of ethanol and 1 mL of water. Water (100 μL) was used to rinse the reaction tube and passed through the cartridge. The column was washed with 200 μL of water, blown dry with nitrogen gas, then the product was slowly eluted dropwise with 200 μL of ethanol into a clean 1.5 mL microfuge tube. The volume of eluent was reduced under dry nitrogen gas flow to approximately 50 μL, diluted into 2 mL of normal saline (Hospira, Lake Forest, IL) and sterile filtered. This purification procedure was confirmed to effectively remove [^68^Ga]­Ga-colloid (see Figure S1).

### Distribution Coefficient (Log *D*) Lipophilicity Assay

[^68^ Ga]­Ga-NODAGA-Pr (962 kBq/26 μCi) or [^68^Ga]­Ga-Pr (740 kBq/20 μCi) in 30 μL 1 M sodium acetate buffer (pH 5.0) was added to an Eppendorf tube containing presaturated phosphate-buffered saline (PBS) pH 7.4 (200 μL) and presaturated 1-octanol (200 μL). The mixture was stirred at room temperature for 10 min in an Eppendorf ThermoMixer at 900 rpm, then centrifuged for 5 min at 1000 rcf. A 50 μL aliquot of the 1-octanol phase (top layer) was collected. The radioactivity in the 50 μL octanol sample was measured using a gamma counter, scaled to the total volume of the octanol phase (×4). All experiments were performed in triplicate. Log *D* was calculated using the equation:

$$\log\left(\frac{\%{\mathrm{Activity}}_{\mathrm{octanol}}}{100\%-\%{\mathrm{Activity}}_{\mathrm{octanol}}}\right)$$

### Stability Assays

[^68^ Ga]­Ga-Pr (629 kBq/17 μCi) or [^68^Ga]­Ga-NODAGA-Pr (888 kBq/24 μCi) in 50 μL 1 M sodium acetate buffer (pH 5.0) was added to 100 μL mouse serum in an Eppendorf tube. The mixture was incubated at 37 °C (600 rpm, Eppendorf ThermoMixer). Aliquots (1.5 μL) were collected at 15, 30, 60, 120, and 360 min for radioTLC analysis (developed with 0.1 M sodium citrate buffer, pH 5.0). All experiments were performed in triplicate.

Additional stability assays were performed with [^68^Ga]­Ga-NODAGA-Pr only. Approximately 5 MBq (135 μCi) of [^68^Ga]­Ga-NODAGA-Pr were incubated in 0.25 mL of mouse serum at 37 °C for 15, 30, and 60 min. At each time point, the serum proteins were removed by precipitation (750 μL of 3:1 CH_3_CN:CH_3_OH), the supernatant was further clarified by ultrafiltration through a 0.22 μm nylon Spin-X spin filter (5 min @ 15,000 × *g*). Radioactivity in the filtrate was examined using radioHPLC (25–50 μL inj. vol.; 4.6 × 250 mm C-18 column; 0–40% gradient (A: 10 mM NH_4_OAc (pH 5.0), B: CH_3_CN)).

### Cell Culture, Animal Models, and DOTA-PRIT Regimen

The GPA33-expressing human CRC cell line SW1222 was obtained from the Ludwig Institute for Cancer Immunotherapy (New York, NY). SW1222 cells were maintained in IMDM media supplemented with 10% fetal bovine serum, 2 mM glutamine, 100 units/mL penicillin, and 100 units/mL streptomycin. Engineered 293T-C825 cells^13^ and parental HEK 293T cells were maintained in RPMI-1640 medium (Memorial Sloan Kettering Cancer Center [MSKCC] Media Core, New York, NY), with the same supplementation.

Female athymic nude mice (aged 6–8 weeks) were obtained from Envigo (West Lafayette, Indiana, USA). In vivo studies were performed at Weill Cornell Medical College (WCM) under Animal Welfare Assurance Numbers A3290-01 (WCM) or A3311-01 (MSKCC). Procedures were performed according to IACUC (Institutional Animal Care and Use Committee) approved protocols (Cheal lab: WCM #2022-0010; Veach Lab: MSK #00–03-053) in the barrier facility/vivarium under strict supervision by the WCM/MSKCC RARC (Research Animal Resource Center). For the SW1222 subcutaneous tumor model, approximately 5 × 10^6^ SW1222 cells suspended in 50% Matrigel (Corning) were injected subcutaneously into the shoulders of the mice. For the 293T-huC825 subcutaneous tumor model, mice were bilaterally implanted with 3 × 10^6^ 293T-huC825 cells (right shoulder) and 3 × 10^6^ parental 293T control cells (left shoulder), both suspended in 50% Matrigel. *In vivo* imaging and biodistribution studies were initiated when the tumor volume reached 100–300 mm^3^, typically 7–11 d postinoculation.

The DOTA-PRIT system consists of three reagents: a bispecific anti-GPA33/anti-DOTA antibody (anti-huA33-C825 BsAb, 210 kDa), the DOTA-dendron clearing agent CCA α-16-DOTA-Y^3+^ (MW: 9059 Da), and a ^68^Ga-radiohapten. All reagents were prepared as previously described.^3,15,16^ A diagram of the experimental protocol is shown in Figure 1.

**Figure 1:**
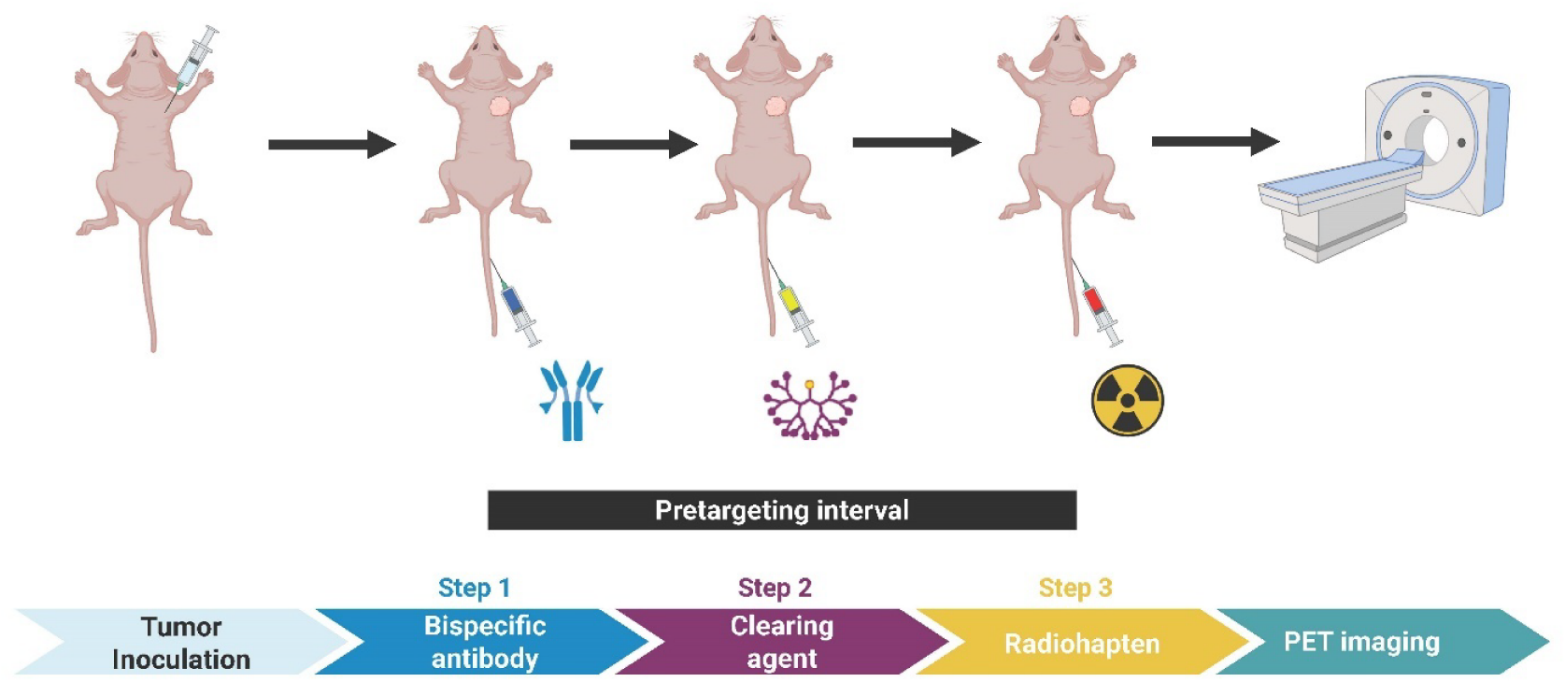
Schematic of *in vivo* studies of the three-step DOTA-PRIT regimen with immunocompromised nude mouse models of human CRC (subcutaneous GPA33-expressing SW1222 xenografts). Bispecific antibody: huA33-C825, clearing agent: CCA α-16-DOTA-Y^3+^, radiohapten: [^68^Ga]­Ga-Pr or [^68^Ga]­Ga-NODAGA-Pr.

SW1222-tumor bearing mice received 0.25 mg (1.19 nmol) of huA33-C825 at *t* = −28 h, followed by 25 μg (2.76 nmol) of the clearing agent at *t* = −4 h, and finally either [^68^Ga]­Ga-Pr or [^68^Ga]­Ga-NODAGA-Pr at *t* = 0 h. Mice bilaterally xenografted with 293T-huC825 and 293T tumors received only the ^68^Ga-radiohapten. All reagents were administered intravenously via the tail vein.

### MicroPET/CT Imaging

PET/CT imaging was performed using the Inveon microPET/CT scanner (Siemens). Mice bearing subcutaneous SW1222 xenografts were injected intravenously with 6.0 MBq (130 pmol) of either GPA33-pretargeted [^68^Ga]­Ga-Pr or [^68^Ga]­Ga-NODAGA-Pr. Mice bearing bilateral 293T-huC825 + 293T xenografts received [^68^Ga]­Ga-NODAGA-Pr only. Immediately prior to scanning, animals were anesthetized with 2% isoflurane and positioned prone. Static images were acquired 1–2 h postinjection (p.i.) using a 15 min scan. To further evaluate ^68^Ga-radiohapten distribution, an *ex vivo* organ biodistribution study was conducted immediately following imaging.

### Biodistribution Study

To more extensively characterize the *in vivo* behavior of DOTA-PRIT with [^68^Ga]­Ga-NODAGA-Pr, we conducted serial biodistribution studies of GPA33-pretargeted [^68^Ga]­Ga-NODAGA-Pr at 5-, 15-, 30-, and 60 min p.i. in groups of SW1222-tumor bearing mice. Cohorts of animals (*n* = 4–5) were euthanized at each time point, and organs of interest were harvested, blotted dry, and weighed in tubes. The radioactivity in each tissue was measured with a gamma counter (PerkinElmer 1480 Wizard 3), corrected for isotope decay, and calculated as percent injected activity per gram (%IA/g).

### Statistical Analysis

All experimental data are presented as mean ± standard deviation. Statistical analyses and nonlinear curve fitting were performed using GraphPad Prism software version 10.1.2. Differences in tissue uptake between groups were assessed using multiple unpaired *t*-tests, with a *P* < 0.05 considered statistically significant.

## Results

### Synthesis of NODAGA-Pr

The synthesis route of NODAGA-Pr is shown in Figure 2A. NODAGA-Pr (15.1 mg, 41%) was obtained as a white foam after lyophilization of the appropriate fractions. ^1^H NMR (600 MHz, D_2_O) δ 7.15–7.25 (m, 4 H), 3.94–3.91 (m, 1 H), 3.89–3.51 (m, 26 H), 3.45–2.81 (m, 24 H), 2.5–2.35 (m, 12 H), 2.20–2.18 (m, 1 H), 2.07–2.03 (m, 1 H), 1.97–1.94 (m, 1 H). We observed two isomers in liquid chromatography–mass spectrometry (LC–MS) with the ratios: 18% and 82%. The minor isomer elutes at 3.02 min, and the major at 3.08 min (total method run time: 8 min). MS (ESI^+^) *m*/*z* calculated for C_49_H_77_LuN_10_O_19_S [M + H]^+^ 1317.45, [M + 2H]^2+^ 659.23, found 659.35.

**Figure 2:**
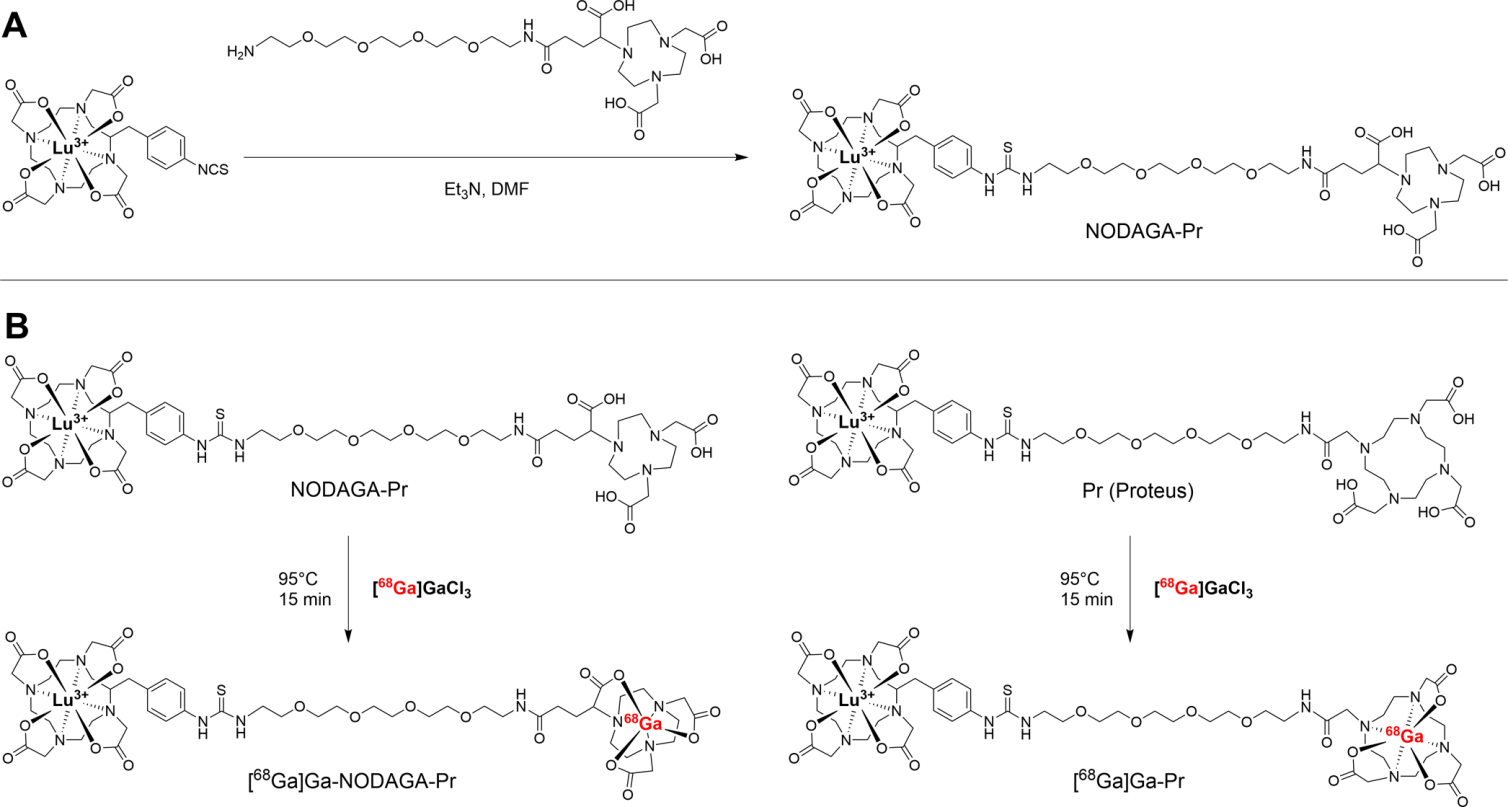
Synthetic route to radiohapten precursor NODAGA-Pr. Lu = nonradioactive Lu-175 (**A**). ^68^Ga-radiohapten radiosynthesis scheme. Lu = nonradioactive Lu-175 (**B**).

### Radiosynthesis and In Vitro Characterization of [^68^Ga]­Ga-NODAGA-Pr and [^68^Ga]­Ga-Pr

The radiosynthesis scheme for ^68^Ga-radiohaptens is illustrated in Figure 2B. Radiolabeling of NODAGA-Pr or Pr with GalliaPharm generator-eluted ^68^Ga was highly efficient (molar activity (*A*_*M*_) at end of synthesis: 70 MBq/nmol (not decay corrected); RCY: >98%; RCP: 98%). RadioHPLC and/or radioTLC of both crude and purified preparations confirmed complete radiolabeling with no detectable free ^68^Ga (radioHPLC: major isomer *R*_*f*_ = 8.1 min, 99+% conversion). A representative radioTLC of crude ^68^Ga-radiosynthesis reaction for [^68^Ga]­Ga-NODAGA-Pr is shown in Figure 3A,B. The maximum observed *A*_*M*_ was 140 MBq/nmol (RCY: >98%; RCP: 98%, final NODAGA-Pr concentration ≥2.5 μM) in ligand mass titration studies, and low conversion was observed with 0.2 nmol of precursor (final NODAGA-Pr concentration ∼0.5 μM) (Figure S2A). Octanol–water (pH 7.4 PBS buffer) distribution coefficients (log *D*_o/w_) were measured using a lipophilicity assay. ^68^Ga-radiohaptens were highly hydrophilic, as summarized in Table 1. The predicted Log *P*_o/w_ values (MLOGP and SILICOS-IT), derived using the web-based *in silico* tool SwissADME^17^ closely align with the experimental values obtained empirically from the lipophilicity assay.

**Table 1:** Select ^68^Ga-Radiohapten Physicochemical and Pharmacokinetic Properties

				Predicted Log *P* _o/w_				
^68^Ga-radiohapten	MW	Calculated molecular charge^c^	Log *D*_7.4_ *n* = 3	MLOGP	SILICOS-IT	Blood half-life (min)	Liver uptake at 1 h p.i. (%IA/g)	Kidney uptake at 1 h p.i. (%IA/g)
[^68^Ga]Ga-NODAGA-Pr	1316.22^a^	–1	–5.09 ± 0.16	–5.06	–4.26	9.2^b^	0.23 ± 0.03 (*n* = 4)	1.85 ± 0.45 (*n* = 4)
[^68^Ga]Ga-Pr	1345.27^a^	–1	–5.22 ± 0.14	–5.59	–5.38	7.8^b^	0.87 ± 0.49 (*n* = 5)	3.18 ± 2.40 (*n* = 5)

a No ^68^Ga, all carboxylate arms of the Lu-complexed DOTA are deprotonated.

b Calculated using nonlinear fit to one phase decay with the following constraints: Y0 > 10 and Plateau >0; [^68^Ga]­Ga-NODAGA-Pr *R* ^2^ = 0.79, [^68^Ga]­Ga-Pr *R* ^2^ = 0.70.

c Predicted ionic behavior under physiologic conditions when gallium-labeled.

**Figure 3:**
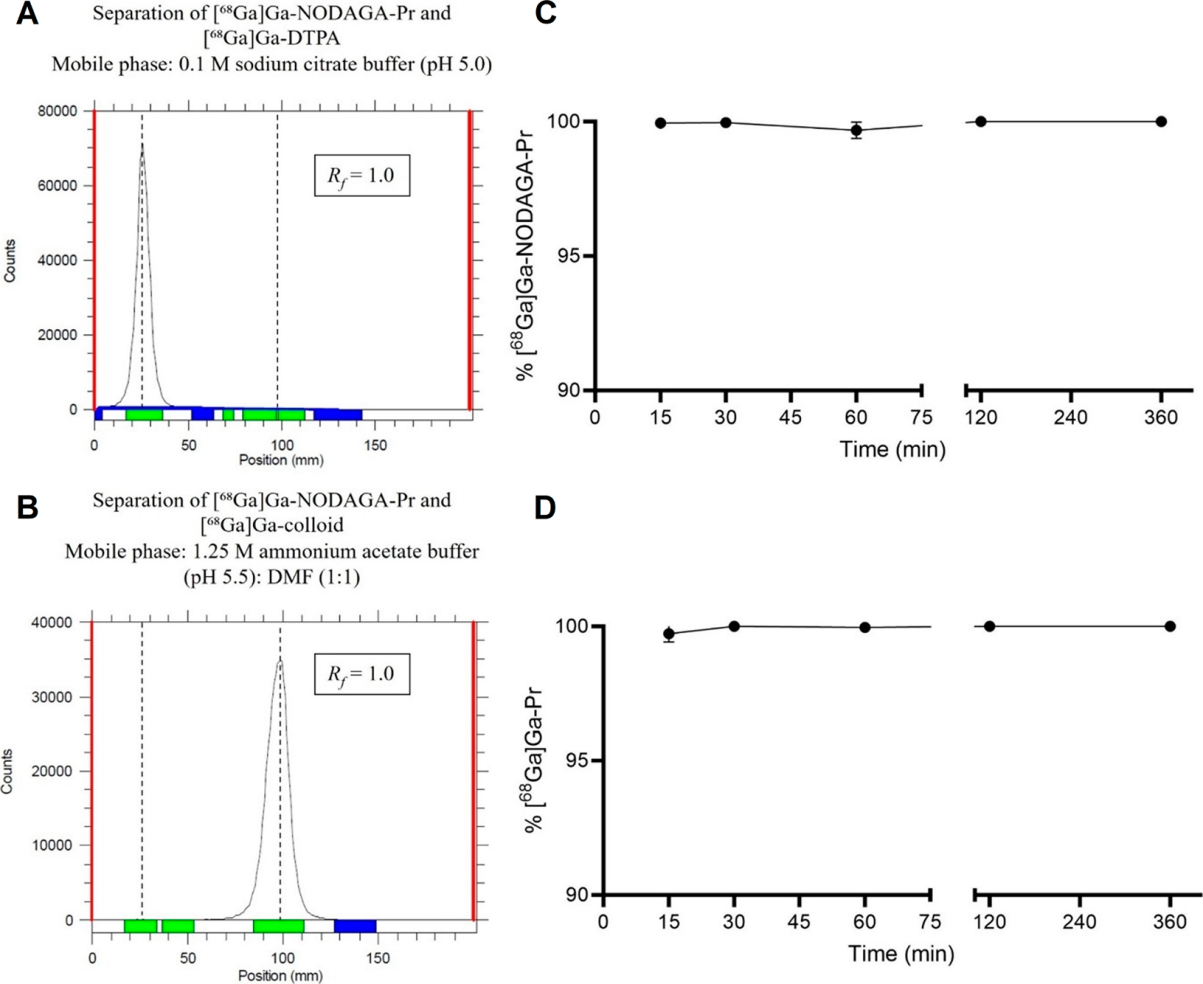
Representative radioTLC of crude ^68^Ga-radiosynthesis reaction for [^68^ Ga]­Ga-NODAGA-Pr (**A and B**). RadioTLC developed with 0.1 M sodium citrate buffer (pH 5.0). Product [^68^Ga]­Ga-NODAGA-Pr remains at baseline (*R_f_* = 0.0) while minimal unreacted ^68^Ga, as [^68^Ga]­Ga-DTPA, was observed at the solvent front (*R_f_* of 1.0), confirming high radiolabeling efficiency (>99%) (**A**). RadioTLC developed with 1.25 M ammonium acetate buffer (pH 5.5): DMF (1:1). [^68^Ga]­Ga-colloid remains at the baseline, while [^68^Ga]­Ga-NODAGA-Pr migrated to an *R_f_* of 1.0. No [^68^Ga]­Ga-colloid was detected (**B**). See also Figure S1. *In vitro* stability of ^68^Ga-radiohaptens in mouse serum at 37 °C (**C** & **D**). Percent intact radiohapten was quantified by radioTLC developed with 0.1 M sodium citrate buffer (pH 5.0). Data is presented as average ± standard deviation; *note*: some error bars are smaller than the symbols.

*In vitro* stability studies in mouse serum demonstrated that both [^68^Ga]­Ga-NODAGA-Pr and [^68^Ga]­Ga-Pr remained intact over 6 h of incubation at 37 °C, with no degradation of at any time point (Figure 3C,D). Additional mouse serum stability studies of [^68^Ga]­Ga-NODAGA-Pr, assessed by radioHPLC, revealed no detectable changes in the radiograms and negligible transfer to serum proteins. These findings suggest no significant demetalation within 1 h and minimal serum-protein binding of radioactivity (Figure S2B).

### Pharmacokinetics and Biodistribution of [^68^Ga]­Ga-NODAGA-Pr and [^68^Ga]­Ga-Pr

Purified ^68^Ga-radiohaptens were administered to healthy mice to evaluate their blood pharmacokinetics and tissue biodistribution at 1 h p.i. (Figures 4 and S2). Both [^68^Ga]­Ga-NODAGA-Pr and [^68^Ga]­Ga-Pr exhibited rapid blood clearance, with half-lives ranging from 7.8 to 9.2 min (Table 1 and Figure S3). No significant differences in tissue uptake were observed between the two radiotracers, except in the liver, where [^68^Ga]­Ga-Pr showed approximately 4-fold higher uptake compared to [^68^Ga]­Ga-NODAGA-Pr (0.87 ± 0.49%IA/g vs 0.23 ± 0.03%IA/g; *P* < 0.05). Overall, these findings indicate that both ^68^Ga-radiohaptens possess rapid systemic clearance and low nonspecific uptake in healthy tissue—favorable characteristics for *in vivo* imaging applications.

**Figure 4:**
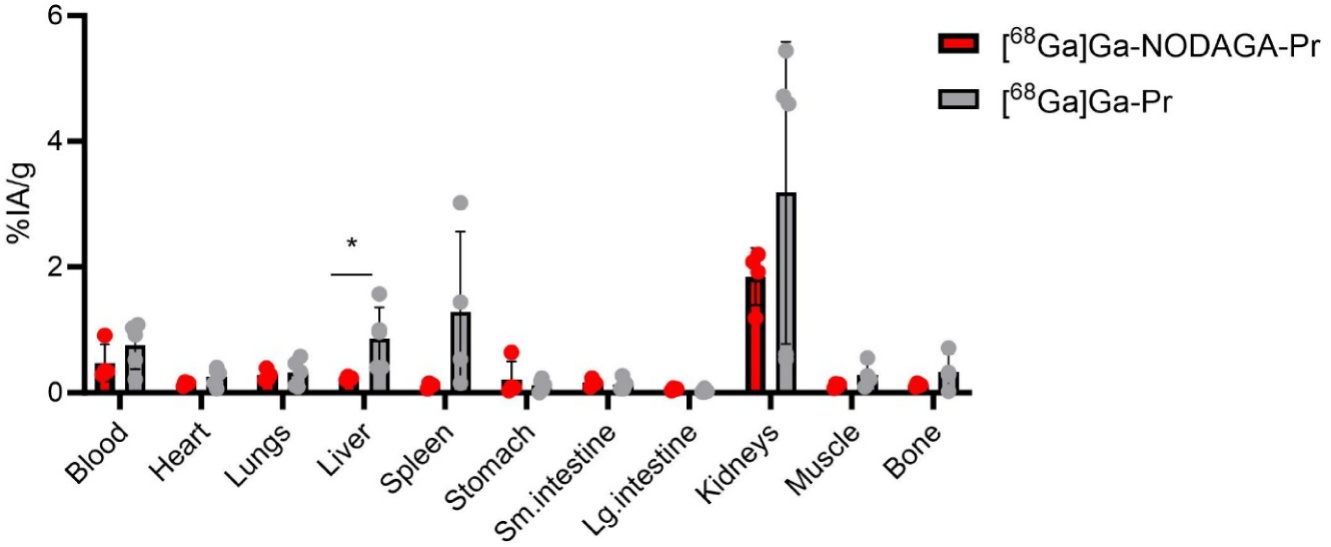
Biodistribution of ^68^Ga-radiohaptens (2.0–2.6 MBq/54–70 μCi, 1 nmol; *A_M_* *=* 2.0–2.6 MBq/nmol at time of injection) in healthy mice at 1 h p.i. (*n* = 4–5). Data is presented as average ± standard deviation. *P < 0.05. Numerical data in tabular form are given in the Supporting Information, Table S1.

### MicroPET/CT Imaging of GPA33-Pretargeted ^68^Ga-Radiohaptens

MicroPET/CT imaging was conducted to assess the *in vivo* tumor targeting efficiency of GPA33-pretargeted [^68^Ga]­Ga-NODAGA-Pr and [^68^Ga]­Ga-Pr in SW1222 xenograft-bearing nude mice (*n* = 3/group). Representative microPET images acquired 2 h p.i. are shown in Figure 5A–D. These images clearly demonstrated focal accumulation of GPA33-pretargeted ^68^Ga-radiohaptens in this CRC model, with distinct tumor delineation coregistered anatomically with CT (Figure 5A,C). In contrast, the nonpretargeted controls (*n* = 1) exhibited negligible tumor uptake, highlighting the specificity of the DOTA-PRIT strategy (Figure 5B,D).

**Figure 5:**
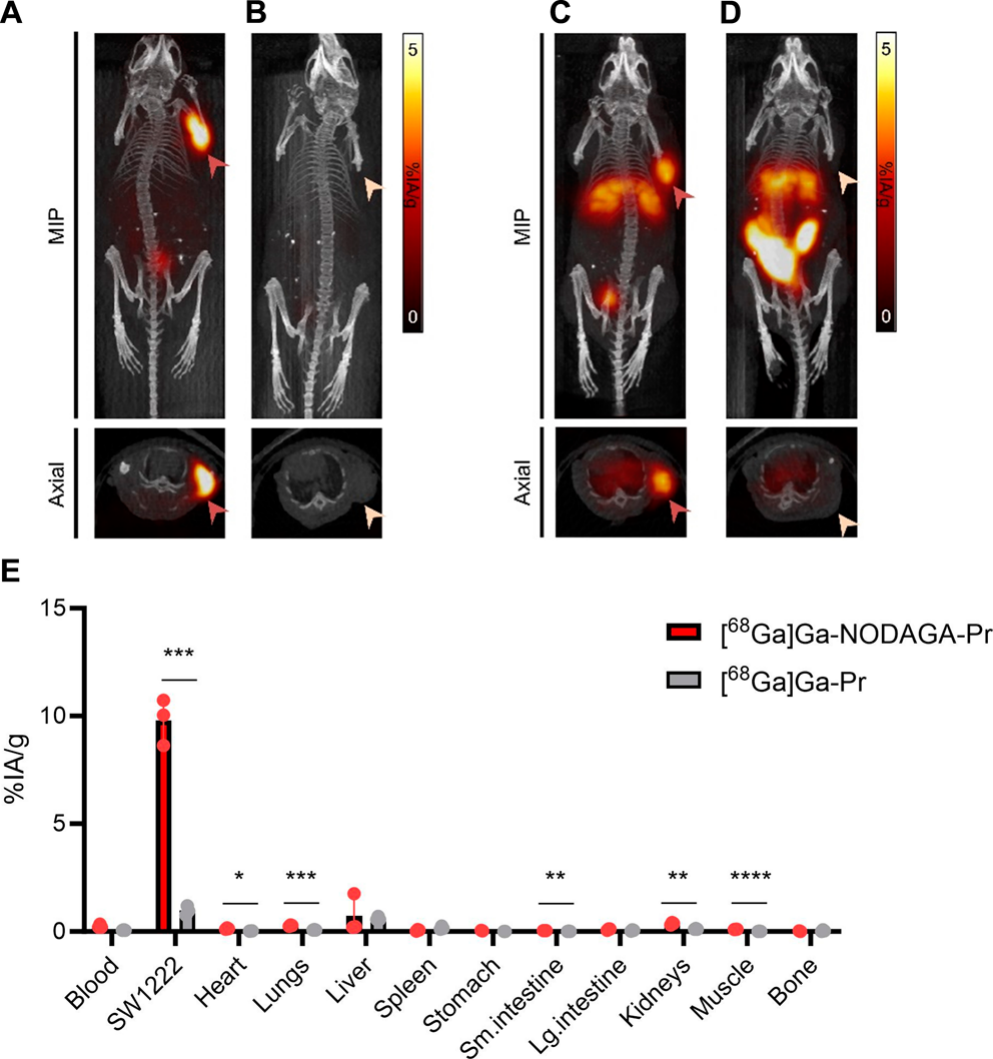
Representative microPET/CT imaging of SW1222-tumor bearing mice at 2 h p.i. of GPA33-pretargeted [^68^Ga]­Ga-NODAGA-Pr (**A**), [^68^Ga]­Ga-NODAGA-Pr only (**B**), GPA33-pretargeted [^68^Ga]­Ga-Pr (**C**), and [^68^Ga]­Ga-Pr only (**D**). For both radiohaptens, 6.0 MBq/162 μCi (130 pmol, *A_M_* *=* 46.2 MBq/nmol at time of injection) was administered intravenously by tail vein. PET images acquired 2 h after tracer injection show the *in vivo* accumulation of pretargeted ^68^Ga-radiohaptens in SW1222 human CRC xenografts in comparison with the accumulation of the nonpretargeted ^68^Ga-radiohaptens in SW1222. *Ex vivo* serial biodistribution studies of ^68^Ga activity performed immediately after PET imaging. (**E**) Data is presented as average ± standard deviation. *P < 0.05; **P < 0.01; ***P < 0.001; ****P < 0.0001. Numerical data in tabular form are given in Table S2. MIP: maximum projection image.

These imaging findings were corroborated by *ex vivo* biodistribution studies performed immediately following imaging (Figure 5E). GPA33-pretargeted [^68^Ga]­Ga-NODAGA-Pr showed robust tumor uptake (9.80 ± 1.07%IA/g) and low background activity in normal tissues. The highest normal tissue uptake was observed in the liver (0.72 ± 0.90%IA/g), with notably low accumulation in the kidneys (0.37 ± 0.06%IA/g). In contrast, pretargeting with [^68^Ga]­Ga-Pr resulted in minimal tumor uptake (<1%IA/g), consistent with the imaging data and indicating poor *in vivo* targeting efficiency. The tumor-to-blood, tumor-to-liver, and tumor-to-kidney ratios for GPA33-pretargeted [^68^Ga]­Ga-NODAGA-Pr were 39.2 ± 7.5, 13.5 ± 9.7, and 26.5 ± 2.8, respectively, indicating higher tumor specificity and minimal off-target accumulation; in contrast, GPA33-pretargeted [^68^Ga]­Ga-Pr exhibited significantly lower ratios of 15.3 ± 2.9, 1.6 ± 0.2, and 8.3 ± 1.6, consistent with suboptimal *in vivo* targeting efficiency.

### MicroPET/CT Imaging of [^68^Ga]­Ga-NODAGA-Pr in the 293T*-*huC825 + 293T Mouse Model

[^68^Ga]­Ga-NODAGA-Pr was identified as the lead ^68^Ga-radiohapten for pretargeted radioimmunodiagnostics. To further evaluate its *in vivo* targeting efficiency, additional microPET/CT imaging studies were performed in mice bearing bilateral 293T-huC825 + 293T xenografts (*n* = 4). Representative microPET images acquired 1 h p.i. are shown in Figure 6A. The *in vivo* imaging (Figure 6A) and *ex vivo* biodistribution data (Figure 6B) demonstrate that [^68^Ga]­Ga-NODAGA-Pr selectively and effectively targeted membrane-bound huC825, with high uptake in 293T-huC825 tumors (8.56 ± 0.18%IA/g), and negligible retention in control 293T tumors (0.07 ± 0.02%IA/g; *P* < 0.0001).

**Figure 6:**
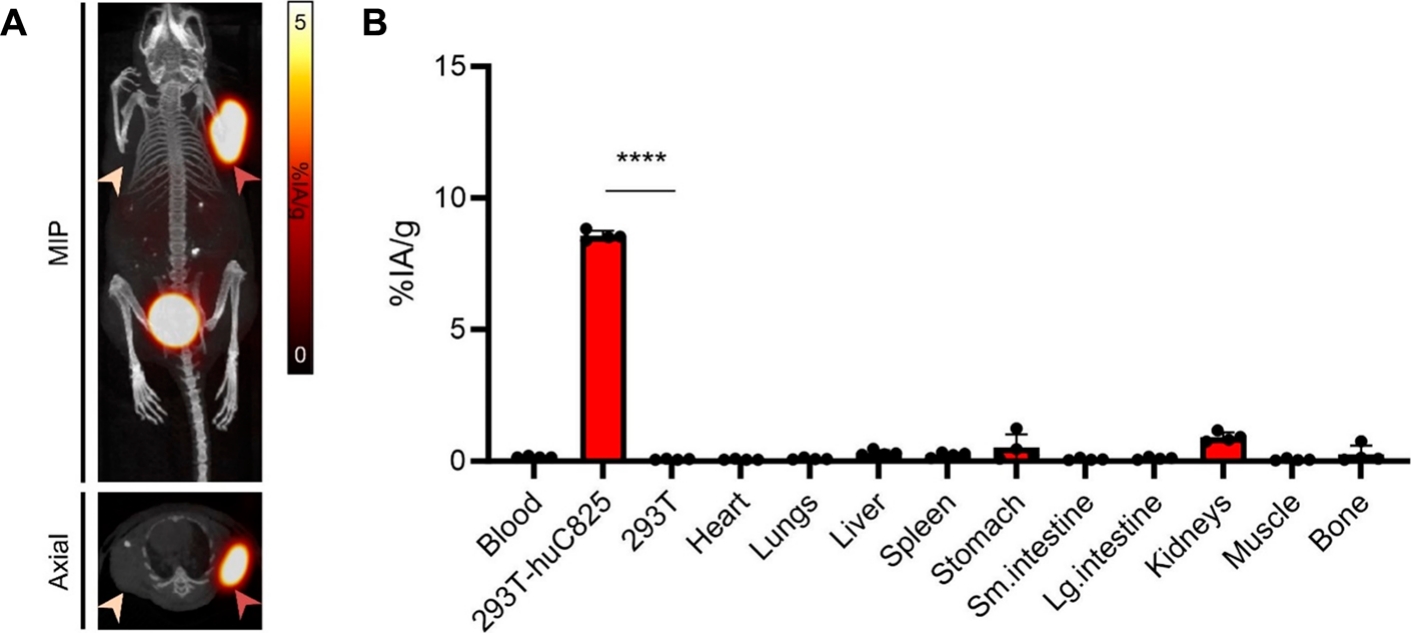
Representative microPET/CT imaging of mice bearing bilateral 293T-huC825/293T xenografts (orange and yellow arrows, respectively) at 1 h p.i. of [^68^Ga]­Ga-NODAGA-Pr 6.0 MBq/162 μCi (130 pmol, *A_M_* *=* 46.2 MBq/nmol at time of injection) administered intravenously. (**A**) *Ex vivo* serial biodistribution studies of ^68^Ga activity performed immediately after PET imaging (*n* = 4). (**B**) Data is presented as average ± standard deviation. ****P < 0.0001. Numerical data in tabular form are given in the Supporting Information, Table S3. MIP: maximum projection image.

The tumor-to-blood, tumor-to-liver, and tumor-to-kidney ratios for [^68^Ga]­Ga-NODAGA-Pr in 293T-huC825 tumors were 54.4 ± 5.0, 26.8 ± 4.3, and 9.4 ± 1.0, respectively, further confirming the high specificity and favorable biodistribution profile.

### MicroPET/CT Imaging of [^68^Ga]­Ga-NODAGA-Pr in the 293T-huC825/293T Mouse Model

[^68^Ga]­Ga-NODAGA-Pr was identified as the superior ^68^Ga-radiohapten for pretargeted radioimmunodiagnosis. To further evaluate its *in vivo* targeting efficiency, additional microPET/CT imaging studies were performed in mice bearing bilateral 293T-huC825 + 293T xenografts (*n* = 4). Representative microPET images acquired 1 h p.i. are shown in Figure 6A. The *in vivo* imaging (Figure 6A) and *ex vivo* biodistribution data (Figure 6B) demonstrate that [^68^Ga]­Ga-NODAGA-Pr selectively and effectively targeted membrane-bound huC825, with high uptake in 293T-huC825 tumors (8.56 ± 0.18%IA/g), and negligible retention in control 293T tumors (0.07 ± 0.02%IA/g; *P* < 0.0001).

The tumor-to-blood, tumor-to-liver, and tumor-to-kidney ratios for [^68^Ga]­Ga-NODAGA-Pr in 293T-huC825 tumors were 54.4 ± 5.0, 26.8 ± 4.3, and 9.4 ± 1.0, respectively, further confirming the high specificity and favorable biodistribution profile of this ^68^Ga-radiohapten.

### Serial Biodistribution Studies of GPA33-Pretargeted [^68^Ga]­Ga-NODAGA-Pr

To further characterize the kinetics of tumor uptake and tissue clearance, serial *ex vivo* biodistribution experiments were performed at 5, 15, 30, and 60 min p.i. of GPA33-pretargeted [^68^Ga]­Ga-NODAGA-Pr (4.0 MBq/108 μCi, 67 pmol) in SW1222-tumor bearing mice (*n* = 4/time point). As shown in Figure 7A,B, the data revealed rapid tumor targeting accompanied by efficient renal clearance. By 60 min p.i., the tumor uptake was approximately 10%IA/g (9.73 ± 1.50%IA/g) with minimal accumulation in normal tissues, including blood (1.18 ± 0.16%IA/g), liver (0.57 ± 0.07%IA/g), and kidneys (0.95 ± 0.14%IA/g). Tumor-to-blood ratios increased progressively from 5 to 30 min, reaching a plateau between 30 and 60 min (5 min: 1.3 ± 0.3, 15 min: 3.3 ± 1.0, 30 min: 9.5 ± 0.6, 60 min: 8.3 ± 0.8), indicating rapid and specific tumor localization of GPA33-pretargeted [^68^Ga]­Ga-NODAGA-Pr.

**Figure 7:**
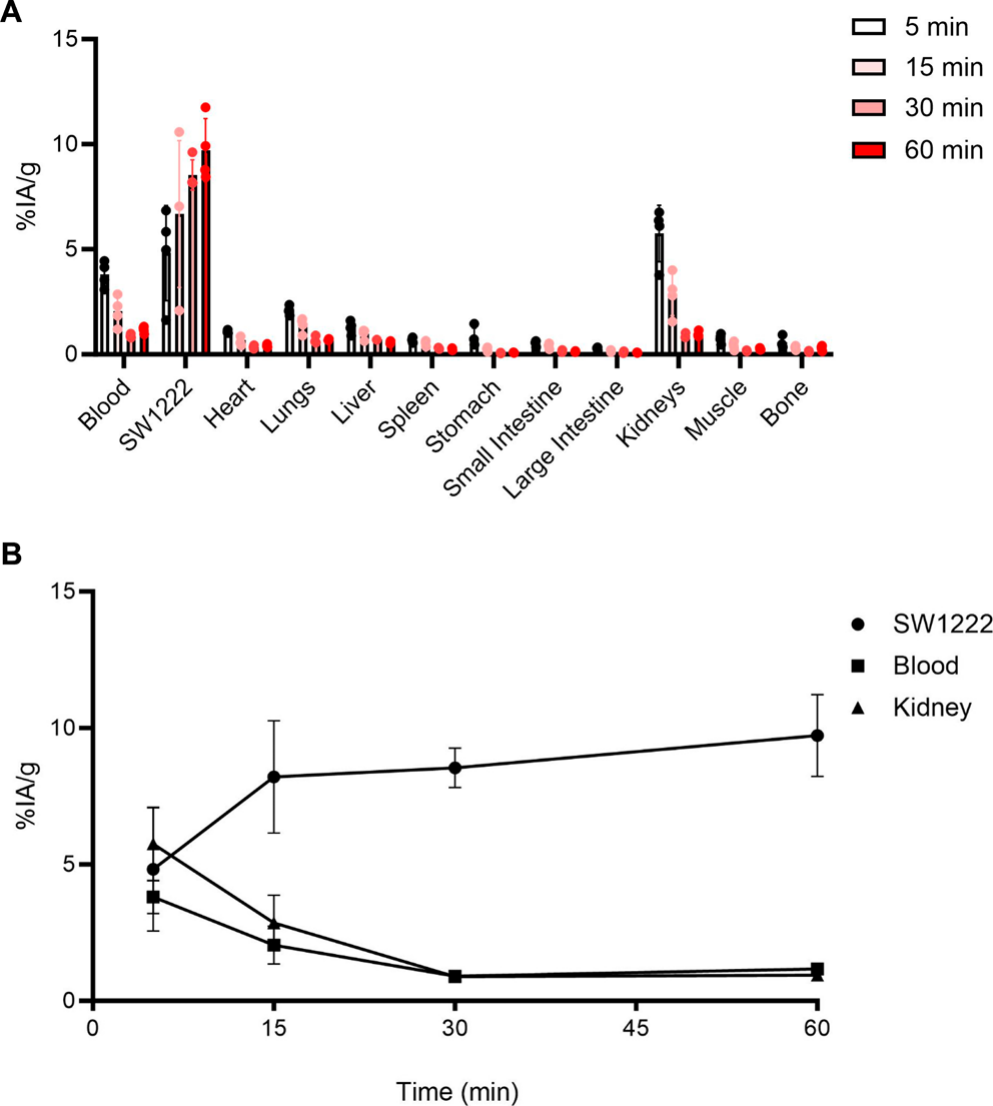
*Ex vivo* serial biodistribution studies conducted from 5 to 60 min p.i. to assess the distribution of ^68^Ga activity in various tissues following GPA33-pretargeting of [^68^Ga]­Ga-NODAGA-Pr in SW1222-tumor bearing mice. Mice (*n* = 4/group) were administered 4.0 MBq/108 μCi (67 pmol, *A_M_* *=* 59.7 MBq/nmol at time of injection) of [^68^Ga]­Ga-NODAGA-Pr. (**A**) Time-activity curves for SW1222 tumor, blood, and kidney showing rapid tumor accrual and excretion from nontargeted critical compartments. (**B**) Data is presented as average ± standard deviation. Numerical data in tabular form are given in the Supporting Information, Table S4. *note*: some error bars are smaller than the symbols.

## Discussion

The successful clinical adoption of PRIT will be accelerated with high-quality surrogate diagnostic radiohaptens. In this context, “high-quality” refers not only to strong molecular and targeting performance but also to practical features such as a radiotracer labeled with a PET radionuclide that is widely accepted in radiopharmacy, kit-friendly radiochemistry, and favorable radiation-safety characteristics. Our goal was to develop and validate a ^68^Ga-DOTA-based pretargeted radioimmunodiagnosis strategy suitable for routine clinical use. To this end, we evaluated two candidate ^68^Ga radiohaptens: [^68^Ga]­Ga-NODAGA-Pr and [^68^Ga]­Ga-Pr.

Using a synthetic approach analogous to that of Pr,^3^ we prepared the NODAGA-Pr precursor in high purity and acceptable yield. Both precursors were efficiently radiolabeled with generator-produced ^68^Ga, yielding products with high molar activity, high purity, and reasonable radiochemical yield. Neither tracer showed evidence of demetalation in mouse serum over 6 h at physiological temperature (Figure 3C,D), supporting their progression to *in vivo* studies.

Although our earlier benzylDOTA radiohapten precursor performs well with ^177^Lu and ^86^Y, its affinity for C825 decreases by orders of magnitude when complexed with ^68^Ga, rendering it unsuitable for *in vivo* tumor pretargeting.^2,18^ Similarly, the original Pr hapten, which contains a DO3A chelator, showed low tumor uptake (<1%IA/g at 2 h p.i.) in anti-GPA33 DOTA-PRIT studies (Figure 5). Because [^68^Ga]­Ga-Pr and [^68^Ga]­Ga-NODAGA-Pr are expected to have similar affinity for C825 (as binding is governed by the ^175^Lu-DOTA portion of Pr), we hypothesize that the poor performance of [^68^Ga]­Ga-Pr arises from a lower effective concentration of intact radioligand available for tumor targeting. Future studies, including radioligand binding assays and *in vivo* metabolite analysis, will be important to confirm this hypothesis and identify potential pathways of radiometal loss.

To address these limitations, we replaced DO3A with the clinically established NODAGA chelator. NODAGA has demonstrated excellent performance in multiple clinical radiopharmaceuticals, including ^68^Ga-NODAGA-JR11 and ^68^Ga-NODAGA-NM-01 formats.^19,20^ The resulting radiohapten, [^68^Ga]­Ga-NODAGA-Pr, showed markedly improved *in vivo* behavior. In two distinct pretargeting systems, it exhibited rapid renal clearance, low background retention, and high tumor uptake—properties that make it a strong candidate for imaging tumor-localized BsAbs and huC825-expressing engineered cells.^14^ Its biodistribution and pharmacokinetics were also consistent with those of other Pr-based radiohaptens, such as [^111^In]­In-Pr and [^225^Ac]­Ac-Pr.^3^ These findings support the use of anti-GPA33 DOTA-PRIT with [^68^Ga]­Ga-NODAGA-Pr PET/CT as a rapid and sensitive method to delineate CRC tumor burden. However, because these studies were limited to a single xenograft model (SW1222), validation in a second, independent GPA33-positive CRC model is warranted to confirm these results.

Our studies highlight the importance of chelator selection in probe design. Although DOTA-based chelators (including DO3A derivatives) are widely used for ^68^Ga radiolabeling, alternative chelators can offer significant advantages in terms of radiosynthesis and image contrast in radiotheranostic applications.^21^ Ga-NODAGA complexes exhibit higher thermodynamic stability constants and faster coordination kinetics than Ga-DO3A complexes.^7^ In contrast, the slower coordination kinetics of DO3A allow a greater fraction of unchelated gallium­(III) during labeling, possibly leading to hydrolysis to Ga­(OH)_3_ and formation of colloidal gallium.^22^ Notably, the colloid formation observed during [^68^Ga]­Ga-Pr radiosynthesis (Figure S1) suggested instability. These differences, along with the susceptibility of Ga-DO3A complexes to transchelation by apo-transferrin,^21^ likely contribute to the poor tumor localization of [^68^Ga]­Ga-Pr, despite its *in vitro* stability in mouse serum. The higher liver uptake observed for [^68^Ga]­Ga-Pr relative to [^68^Ga]­Ga-NODAGA-Pr at 1 h p.i. in normal mice (Figure 4) further suggests reduced kinetic inertness and increased *in vivo* demetalation.

Serial biodistribution studies of GPA33-pretargeted [^68^Ga]­Ga-NODAGA-Pr demonstrated rapid and efficient tumor localization, with peak uptake achieved within 15 min p.i. and optimal tumor-to-blood, tumor-to-liver, and tumor-to-kidney ratios reached by 30 min p.i. (9.5 ± 0.6, 12.2 ± 0.5, and 9.5 ± 0.8, respectively). ^68^Ga radiohapten PET imaging was informative at both 1 and 2 h p.i., however, the latter time point had the advantage of providing enough time for Ga-68 tracer clearance resulting in a much lower confounding bladder signal. These kinetics are well matched to the physical half-life of ^68^Ga (*t*_1/2_ = 1.1 h), supporting its suitability for clinical PET imaging.

Collectively, these findings support several promising applications for [^68^Ga]­Ga-NODAGA-Pr. It could serve as a diagnostic companion to β-emitting radiohaptens such as [^177^Lu]­Lu-DOTA in upcoming DOTA-PRIT clinical trials or facilitate translation of α-emitting radiohaptens such as [^225^Ac]­Ac-Pr. Further evaluation is warranted using the self-assembling and disassembling (SADA) BsAb platform,^23^ a next-generation two-step pretargeting system that does not require a separate clearing agent for optimal radiohapten delivery. The SADA platform delivering [^177^Lu]­Lu-DOTA to GD2- or CD38-expressing cancers is currently in clinical trials (NCT05130255, NCT05994157), and may benefit from a ^68^Ga PET companion diagnostic hapten such as [^68^Ga]­Ga-NODAGA-Pr. Additionally, [^68^Ga]­Ga-NODAGA-Pr is well suited for imaging cellular therapies engineered with the anti-DOTA scFv huC825. For example, Kurtz et al. developed CD19 CAR-T cells (“Thor-cells”) engineered to express huC825 for radiohapten capture in imaging and radioimmunotherapy.^14^ While their study used [^86^Y]­Y-benzylDOTA for PET imaging,^14^ [^68^Ga]­Ga-NODAGA-Pr would serve as a more accessible alternative for monitoring the trafficking, expansion, and tumor infiltration of huC825 reporter-enabled CAR-T cell therapy. Notably, we achieved high tumor uptake of ^68^Ga-activity (8.56 ± 0.18%IA/g) in the 293T-huC825 model despite the relatively low number of binding sites per cells (∼20k^13^), supporting the feasibility of this strategy.

## Conclusion

Here, we report the synthesis and *in vivo* evaluation of [^68^Ga]­Ga-NODAGA-Pr, a novel PET radiohapten designed for ^68^Ga-DOTA-based pretargeted radioimmunodiagnosis. Our findings demonstrated that [^68^Ga]­Ga-NODAGA-Pr immunoPET can accurately and selectively delineate GPA33-positive CRC in a xenograft model. Importantly, we find that [^68^Ga]­Ga-NODAGA-Pr is straightforward to manufacture in a manner similar to existing Ga-68 radiopharmaceuticals from a ready-to-use kit.^24^ This strategy leverages a widely available and clinically accepted PET isotope, which lowers the barrier to regulatory filing and clinical translation. Through our work, we are reminded that a clinically and commercially successful chelator system like ^68^Ga-DO3A may look promising in development, but it is not automatically compatible with any drug system *in vivo*.

In summary, [^68^Ga]­Ga-NODAGA-Pr represents a promising diagnostic tool that integrates well with PRIT applications and offers a practical approach for noninvasive monitoring of engineered cellular immunotherapies.

## Supplementary Material



## Notes

Supplementary Figures (S1–S3): representative radioTLC of [^68^Ga]­Ga-DTPA and detection of [^68^Ga]­Ga-colloid in crude [^68^Ga]­Ga-Pr; *in vitro* characterization of [^68^Ga]­Ga-NODAGA-Pr, including mass titration and serum stability studies; blood pharmacokinetics of [^68^Ga]­Ga-NODAGA-Pr and [^68^Ga]­Ga-Pr in healthy nude mice; Supplementary Tables (S1–S4): tabulated *ex vivo* biodistribution data for [^68^Ga]­Ga-NODAGA-Pr or [^68^Ga]­Ga-Pr in healthy nude mice at 1 h p.i.; tabulated *ex vivo* biodistribution data for GPA33-pretargeted [^68^Ga]­Ga-NODAGA-Pr and GPA33-pretargeted [^68^Ga]­Ga-Pr in SW1222-tumor bearing mice at 1 h p.i.; tabulated *ex vivo* biodistribution data for [^68^Ga]­Ga-NODAGA-Pr in mice bearing bilateral 293T-huC825/293T xenografts at 1 h p.i.; tabulated ex vivo biodistribution data for GPA33-pretargeted [^68^Ga]­Ga-NODAGA-Pr in SW1222-tumor bearing mice at 5–60 min p.i. (PDF)
