# Supplementary material for: Establishment of 68Ga-DOTA-Based Pretargeted Radioimmunodiagnosis
Source: Mol Pharm. 2026 May 19;23(6):3265–75. doi: 10.1021/acs.molpharmaceut.5c01766 (PMC13231412; doi:10.1021/acs.molpharmaceut.5c01766)
Supplement: Supplementary file 1 [file mp5c01766_si_001.pdf]

Establishment of ^{68}Ga-DOTA-Based Pretargeted Radioimmunodiagnosis

Darren R. Veach[a,e], Daniela Burnes Vargas[b], Baharul Islam[g], Sang Gyu Lee[a], Leah Gajecki[a], Brett A. Vaughn[b,g], Guangbin Yang[c], Teja Muralidhar Kalidindi[a], Naga Vara Kishore Pillarsetty[a,e], Niloufar Salehi[g], Ambika P. Jaswal[g], Sayani Saha[g], Alexandre B. Le Roux[a], Hong Xu[d], Hong-fen Guo[d], Ouathek Ouerfelli[c], Nai-Kong V. Cheung[d], Simone Krebs[a,e,f], Steven M. Larson[a,b], Sarah M. Cheal[b,g].*

[a]*Department of Radiology, Memorial Sloan Kettering Cancer Center, New York 10065, United States;*
[b]*Program in Molecular Pharmacology, Memorial Sloan Kettering Cancer Center, New York 10065, United States*
[c]*Organic Synthesis Core Facility, Memorial Sloan Kettering Cancer Center, New York 10065, United States;*
[d]*Department of Pediatrics, Memorial Sloan Kettering Cancer Center, New York 10065, United States;*
[e]*Department of Radiology, Weill Cornell Medicine, New York 10021, United States.*
[f]*Department of Nuclear Medicine, The University of Texas MD Anderson Cancer Center, Houston, Texas 77030, United States*
[g]*Molecular Imaging Innovations Institute, Department of Radiology, Weill Cornell Medicine, New York 10021, United States.*

*Email: smc4002@med.cornell.edu

Supplementary Material

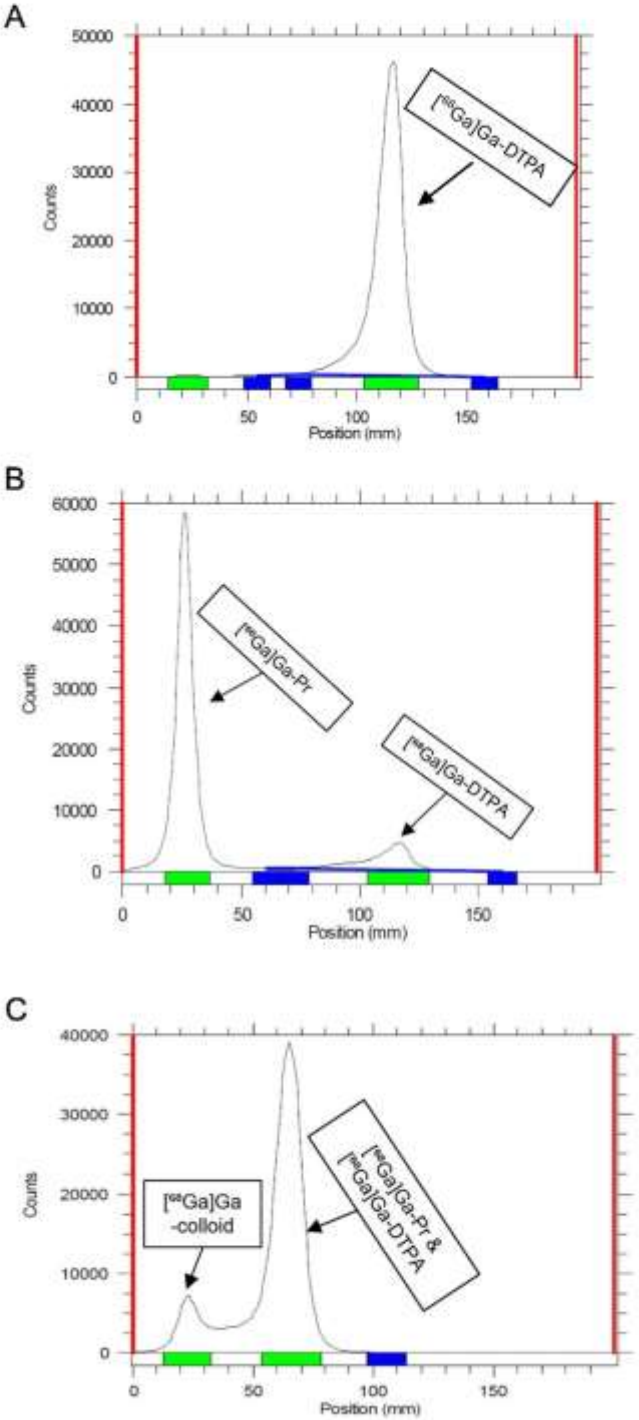

Figure S1. Representative radioTLC of [^{68}Ga]Ga-DTPA and detection of [^{68}Ga]Ga-colloid in crude [^{68}Ga]Ga-Pr. RadioTLC of [^{68}Ga]Ga-DTPA (mobile phase: 0.1 M sodium citrate buffer (pH 5.0)). [^{68}Ga]Ga-DTPA migrates to an *Rf* of 1.0. (**A**) RadioTLC of crude reaction of [^{68}Ga]Ga-Pr (mobile phase: 0.1 M sodium citrate buffer (pH 5.0)). Labeling yield: 89%. (**B**) RadioTLC of crude reaction of [^{68}Ga]Ga-Pr developed with 1.25 M ammonium acetate buffer (pH 5.5):DMF (1:1). [^{68}Ga]Ga-colloid remains at the baseline, while the [^{68}Ga]Ga-Pr and [^{68}Ga]Ga-DTPA migrates to an *Rf* of 1.0. [^{68}Ga]Ga-colloid was detected in the crude [^{68}Ga]Ga-Pr reaction. (**C**)

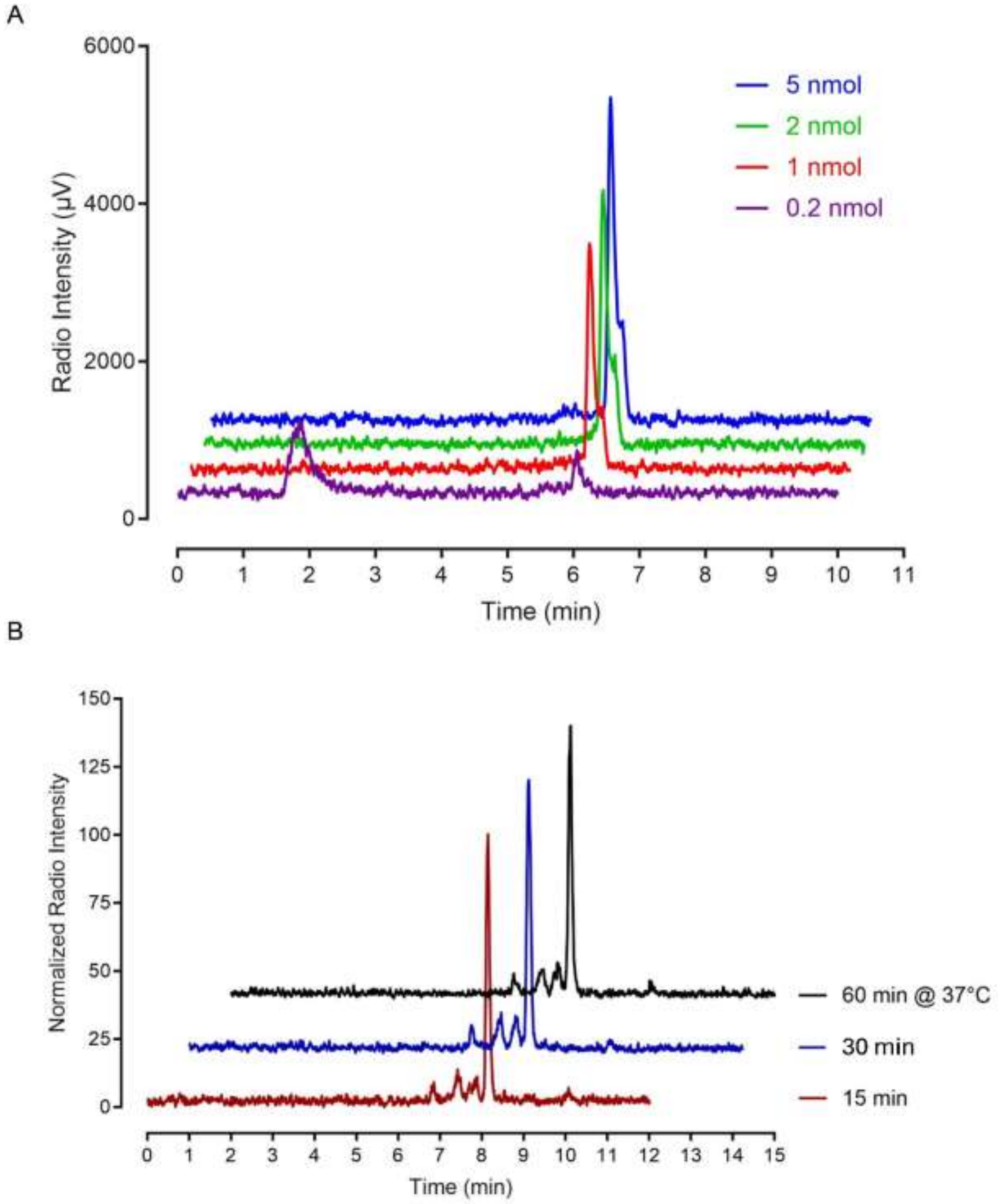

Figure S2. *In vitro* characterization of [^{68}Ga]Ga-NODAGA-Pr. Mass titration studies of the NODAGA-Pr precursor NODAGA-Pr for the radiosynthesis of [^{68}Ga]Ga-NODAGA-Pr. (**A**) *In vitro* stability analysis of [^{68}Ga]Ga-NODAGA-Pr in mouse serum at 37 °C. (**B**)

Table S1. *Ex vivo* biodistribution studies of ^{68}Ga activity in various tissues for [^{68}Ga]Ga-NODAGA-Pr (2.0–2.6 MBq/54–70 µCi, 1 nmol) or [^{68}Ga]Ga-Pr (2.0–2.6 MBq/54–70 µCi, 1 nmol) in healthy nude mice at 1 h p.i. of ^{68}Ga activity. SD: standard deviation

Organ	[^{68}Ga]Ga-NODAGA-Pr %IA/g			[^{68}Ga]Ga-Pr %IA/g		
	Mean	SD	*n*	Mean	SD	*n*
Blood	0.47	0.29	4	0.75	0.37	5
Heart	0.14	0.03	4	0.52	0.14	5
Lungs	0.28	0.09	4	0.32	0.22	5
Liver	0.23	0.03	4	0.87	0.49	5
Spleen	0.11	0.04	4	1.29	1.28	4*
Stomach	0.21	0.29	4	0.12	0.09	5
Sm. Intestine	0.16	0.06	4	0.13	0.09	5
Lg. Intestine	0.05	0.02	4	0.03	0.03	4*
Kidney	1.85	0.45	4	3.18	2.40	5
Muscle	0.11	0.03	4	0.28	0.20	4*
Bone	0.12	0.02	4	0.33	0.29	4*

*a single tissue activity measurement (cpm) was less than background and thus excluded

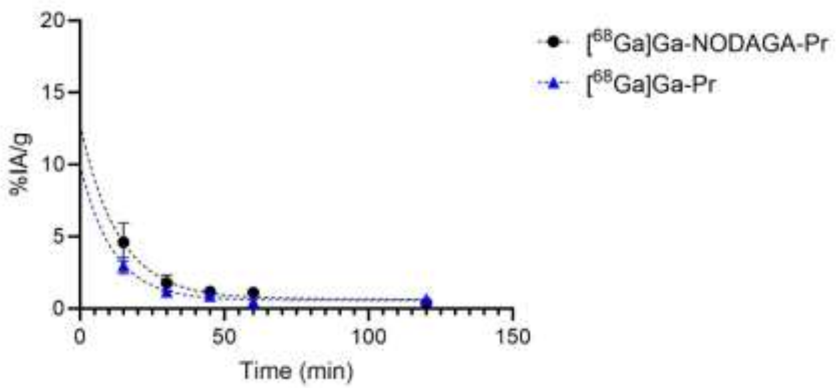

Time (min)	[^{68}Ga]Ga-NODAGA-Pr	*n*	[^{68}Ga]Ga-Pr	*n*
15	4.61 ± 2.30	3	2.97 ± 0.97	3
30	1.77 ± 0.95	3	1.18 ± 0.47	3
45	1.17 ± 0.40	3	0.88 ± 0.47	3
60	1.10 ± 0.42	3	0.48 ± 0.08	3
120	0.38 ± 0.04	3	0.66 ± 0.22	2

Figure S3. Blood pharmacokinetics for [^{68}Ga]Ga-NODAGA-Pr (2.0–2.6 MBq/54–70 µCi, 1 nmol) or [^{68}Ga]Ga-Pr (2.0–2.6 MBq/54–70 µCi, 1 nmol) in healthy nude mice from 15–120 min p.i. of ^{68}Ga activity. Dotted line is nonlinear fit to one phase decay with the following constraints: Y0 > 10 and Plateau > 0; [^{68}Ga]Ga-NODAGA-Pr $t_{1/2}$ = 9.2 min R^2 = 0.79, [^{68}Ga]Ga-Pr $t_{1/2}$ = 7.8 min R^2 = 0.70. Data in table is presented as average ± standard deviation. In some cases, error bars are smaller than the data symbols.

Table S2. *Ex vivo* biodistribution studies of ^{68}Ga activity in various tissues for GPA33-pretargeted [^{68}Ga]Ga-NODAGA-Pr (6.0 MBq/162 µCi, 130 pmol) or GPA33-pretargeted [^{68}Ga]Ga-Pr (6.0 MBq/162 µCi, 130 pmol) in SW1222-tumor bearing mice at 2 h p.i. of ^{68}Ga activity. Data in table is presented as average ± standard deviation.

Organ	GPA33-pretargeted [^{68}Ga]Ga-NODAGA-Pr %IA/g *n* = 3	[^{68}Ga]Ga-NODAGA-Pr only %IA/g *n* = 1	GPA33-pretargeted [^{68}Ga]Ga-Pr %IA/g *n* = 3	[^{68}Ga]Ga-Pr only %IA/g *n* = 1
Blood	0.25 ± 0.08	0.03	0.06 ± 0.02	0.05
SW1222 tumor	9.80 ± 1.07	0.04	0.97 ± 0.22	0.07
Heart	0.13 ± 0.04	0.01	0.03 ± 0.01	0.02
Lungs	0.28 ± 0.04	0.03	0.08 ± 0.01	0.05
Liver	0.72 ± 0.90	0.14	0.61 ± 0.09	1.19
Spleen	0.06 ± 0.04	0.05	0.17 ± 0.07	0.36
Stomach	0.04 ± 0.02	0.01	0.01 ± 0.00	0.07
Sm. Intestine	0.04 ± 0.01	0.01	0.01 ± 0.01	0.04
Lg. Intestine	0.10 ± 0.03	0.16	0.05 ± 0.03	1.70
Kidney	0.37 ± 0.06	0.46	0.12 ± 0.03	0.18
Muscle	0.10 ± 0.01	0.03	0.01 ± 0.01	0.03
Bone	0.01 ± 0.02	0.12	0.04 ± 0.04	0.07

Table S3. *Ex vivo* biodistribution studies of ^{68}Ga activity in various tissues for [^{68}Ga]Ga-NODAGA-Pr (6.0 MBq/162 µCi, 130 pmol) in mice bearing bilateral 293T-huC825/293T xenografts at 1 h p.i. of ^{68}Ga activity. Data in table is presented as average ± standard deviation.

Organ	[^{68}Ga]Ga-NODAGA-Pr %IA/g *n* = 4
Blood	0.16 ± 0.03
293T-huC825	8.56 ± 0.18
293T	0.07 ± 0.02
Heart	0.07 ± 0.02
Lungs	0.09 ± 0.03
Liver	0.32 ± 0.10
Spleen	0.24 ± 0.09
Stomach	0.51 ± 0.51
Sm. Intestine	0.08 ± 0.04
Lg. Intestine	0.11 ± 0.05
Kidney	0.91 ± 0.19
Muscle	0.06 ± 0.04
Bone	0.26 ± 0.34

Table S4. *Ex vivo* biodistribution studies of ^{68}Ga activity in various tissues for GPA33-pretargeted [^{68}Ga]Ga-NODAGA-Pr (4.0 MBq/108 μCi, 67 pmol) in SW1222-tumor bearing mice at 5–60 min p.i. of ^{68}Ga activity. Data in table is presented as average ± standard deviation.

Organ	5 min p.i. %IA/g *n* = 4	15 min p.i. %IA/g *n* = 4	30 min p.i. %IA/g *n* = 4	60 min p.i. %IA/g *n* = 4
Blood	3.80 ± 0.61	2.05 ± 0.70	0.90 ± 0.09	1.18 ± 0.16
SW1222 tumor	4.82 ± 2.26	6.68 ± 3.49	8.54 ± 0.72	9.73 ± 1.50
Heart	1.08 ± 0.07	0.68 ± 0.23	0.32 ± 0.09	0.43 ± 0.07
Lungs	2.09 ± 0.21	1.39 ± 0.35	0.66 ± 0.16	0.66 ± 0.05
Liver	1.28 ± 0.30	0.97 ± 0.23	0.70 ± 0.01	0.57 ± 0.07
Spleen	0.67 ± 0.11	0.53 ± 0.11	0.30 ± 0.03	0.25 ± 0.06
Stomach	0.78 ± 0.47	0.24 ± 0.09	0.06 ± 0.02	0.10 ± 0.02
Sm. Intestine	0.52 ± 0.12	0.37 ± 0.13	0.13 ± 0.05	0.13 ± 0.03
Lg. Intestine	0.26 ± 0.07	0.16 ± 0.05	0.08 ± 0.04	0.09 ± 0.01
Kidney	5.75 ± 1.35	2.86 ± 1.01	0.89 ± 0.13	0.95 ± 0.14
Muscle	0.75 ± 0.23	0.42 ± 0.19	0.17 ± 0.04	0.28 ± 0.06
Bone	0.57 ± 0.25	0.31 ± 0.09	0.13 ± 0.03	0.28 ± 0.12
